# Assessment of bone tissue cytoarchitectonics by 2D ^1^H NMR relaxometry maps

**DOI:** 10.1007/s10867-024-09658-2

**Published:** 2024-06-27

**Authors:** Emese Orban, Zsuzsanna Pap, Remus Sebastian Sipos, Radu Fechete

**Affiliations:** 1 George Emil Palade University of Medicine, Pharmacy, Science, and Technology of Targu Mures, 38 Gheorghe Marinescu Str., Targu Mures 540139, Romania; 2 Technical University of Cluj-Napoca, 28 Memorandumului Str., Cluj-Napoca-Napoca, 400114 Romania

**Keywords:** Osteoporosis, Simvastatin, Fenofibrate, Pore connectivity, ^1^H NMR relaxometry

## Abstract

Bone is a complex tissue that fulfills the role of a resistance structure. This quality is most commonly assessed by bone densitometry, but bone strength may not only be related to bone mineral density but also to the preservation of bone cytoarchitectonics. The study included two groups of rats, ovariectomized and non-ovariectomized. Each group was divided into three batches: control, simvastatin-treated, and fenofibrate-treated. In the ovariectomized group, hypolipidemic treatment was instituted at 12 weeks post ovariectomy. One rat from each of the 6 batches was sacrificed 8 weeks after the start of treatment in the group. The experimental study was performed using a Bruker Minispec mq 20 spectrometer operating at a frequency of 20 MHz, subsequently also performed by ^1^H *T*_2_-*T*_2_ molecular exchange maps. The results were represented by *T*_2_-*T*_2_ molecular exchange maps that showed, comparatively, both pore size and their interconnectivity at the level of the femoral epiphysis, being able to evaluate both the effect of estrogen on bone tissue biology and the effect of the lipid-lowering medication, simvastatin, and fenofibrate, in both the presence and absence of estrogen. *T*_2_-*T*_2_ molecular exchange maps showed that the absence of estrogen results in an increase in bone tissue pore size and interconnectivity. In the presence of estrogen, lipid-lowering medication, both simvastatin and fenofibrate alter bone tissue cytoarchitectonics by reducing pore interconnectivity. In the absence of estrogen, fenofibrate improves bone tissue cytoarchitectonics, the *T*_2_-*T*_2_ molecular exchange map being similar to that of non-osteoporotic bone tissue.

## Introduction

Bones are rigid organs of the skeletal system with multiple functions, such as supporting and protecting internal organs, providing support for muscles and playing a role in the production of blood cells. In terms of cytoarchitectonic organization, bone is divided into compact bone and cancellous bone [1]. The fundamental unit of bone that characterizes compact bone microscopically is the osteon or Haversian system. It consists of a central canal (the Haversian canal) which is surrounded by concentric bone lamellae in which osteocytes, mature bone cells, are embedded in bone spaces called lacunae and communicate with each other via bone canaliculi [2]. The Haversian canal contains the blood vessels and nerves that provide nutrition and innervation to bone. Bone trabeculae, which belong to the cancellous part of the bone, are filamentous structures within the bone with a role in shock absorption and force redistribution [3].

These particularities related to the differentiated structure of the two types of bone tissue explain the different clinical behavior determined by the different cytoarchitectonics of the two bone types [4]. Thus, trabecular bone tissue in the vertebral bodies may produce microfractures followed by vertebral subsidence without acute clinical consequences in the absence of trauma, with structural changes leading to late but chronic clinical signs. In contrast, in cortical bone fractures usually occur posttraumatically and are accompanied by an acute clinical picture [5, 6]. These bone peculiarities also determine a different evolution of the callus formation process, in situations where callus occurs following fracture of healthy or pathological bone tissue [7, 8].

Fracture healing is a complex process that occurs in stages, from an initial phase determined by the appearance of post-fracture hematoma to a phase of primary callus that undergoes a process of bone remodeling, resulting in definitive callus, i.e., actual healing [9]. Within these stages, regardless of the fracture location, the patient's quality of life suffers a degree of impairment, and the callus formation process can be influenced by a number of factors [10, 11], including the patient’s acute or chronic medication [12].

Osteoporosis is a condition characterized by decreased bone density and deterioration of the microarchitecture of bone tissue, which increases the risk of fractures. It is one of the most common skeletal conditions and mainly affects older people, especially postmenopausal women. Osteoporosis can affect any bone in the body, but the most common fractures are those of the femoral neck, spine, and wrist [13, 14]. Several causes and risk factors for osteoporosis are described. Estrogen deficiency for example, in the postmenopausal period, leads to loss of bone density. Another risk factor is old age [15]. In terms of clinical manifestations, osteoporosis is a relatively silent disease, especially in its early stages. People with osteoporosis are at increased risk of fractures; on the other hand, loss of bone density can lead to bone pain and bone deformities [16, 17].

Dyslipidemia is a condition characterized by abnormal levels of lipids in the blood. This includes changes in the concentration of cholesterol and triglycerides. Treatment of dyslipidemia includes lifestyle changes and drug therapy [16, 18]. Lipid-lowering medication is used to control blood lipid levels in order to reduce the risk of cardiovascular disease associated with dyslipidemia. These drugs have various mechanisms of action and can be effective in lowering total cholesterol, LDL (low-density lipoprotein) cholesterol, and triglyceride levels while aiming to increase HDL (high-density lipoprotein) cholesterol levels [19].

Recent studies have suggested that 3-hydroxy-3-methylglutaryl-coenzyme A (HMG-CoA) reductase inhibitors may increase bone mineral density [20]. Patients on statins were more likely to have higher bone mineral density and a lower risk of osteoporosis than patients who have not received this treatment [21]. Studies consider dose dependence when investigating the relationship between statins and osteoporosis [22, 23].

Some experimental studies have shown that fibrates stimulate preosteoblast proliferation and the release of osteoprotegerin, an important inhibitor of osteoclast differentiation, indicating that fibrates may have an antiresorptive effect. At the same time, fibrates stimulate osteoblast differentiation and proliferation, promoting bone matrix formation [24].

## Innovative methods for bone tissue assessment

Porous media magnetic resonance (PMMR) has become an exciting branch of science that enjoys a wide range of applications and opportunities for involvement in multidisciplinary research. Porous media is generally considered to be a material with important structures at length scales larger than atoms, and these structures are essential to the material's properties and performance. For example, fluid transport is an essential property of many porous media, from water movement to chemical reactions in porous catalysts, filtration, gas exchange in lung tissue, and blood and oxygen perfusion in the brain and in cancer growth. Biological tissues have complex structures, and many aspects are similar to inorganic porous media. For example, trabecular bones are porous; their porous structure is important for mechanical integrity. Bone loss and change in its structure are a key element of bone fractures, especially in old age [25]. NMR methods allow the determination of the water content and also the characterization of the pore network by longitudinal *T*_1_ or transverse *T*_2_ relaxation time. Being sensitive to the interaction between spins, which is influenced by the motion of molecules, *T*_2_ is one of the most important NMR parameters measured for porous materials [26].

The aim of the study is to open new perspectives in addressing osteoporosis by allowing the assessment of bone tissue cytoarchitectonics and an approach that targets the potential favorable side effects of a widely prescribed drug in patients at an age at risk of developing osteoporosis. Accumulating complementary evidence showing that lipid-lowering medication may have dual therapeutic potential through multiple methods could change the approach to some chronic diseases [27].

## Material and method

Three rats belonging to each group were selected for this study. The first group consisted of rats that were ovariectomized and after 12 weeks after ovariectomy they were divided into three batches: a control batch, a fenofibrate-treated batch, and a simvastatin-treated batch. Both drugs were administered by gavage in a dose of 10 mg/kgc. The second group consisted of rats aged 16–18 months (human equivalent: females 47–52 years, perimenopausal period), non-ovariectomized, and this group was further divided into three batches, a control batch, a fenofibrate-treated batch, and a simvastatin-treated batch, both administered by gavage at a dose of 10 mg/kgc. The animals were euthanized 8 weeks after the start of treatment in both the fenofibrate and simvastatin batches and in the control batch. After euthanasia, the left femur was removed, and its proximal end was fixated in 10% formalin for study.

The Scientific Research Ethics Committee of the University of Medicine, Pharmacy, Sciences and Technology “George Emil Palade” of Targu Mures approved the experiment and the study protocols according to documents no. 609/19.12.2019. The medical and surgical interventions were performed following the approval of the experiment and study protocols by the Ethics and Deontology Commission of the University of Medicine and Pharmacy of Târgu Mureș, according to documents no. 2/2009, 29/26.06.2012.

The experiments were performed using a Bruker Minispec mq 20 spectrometer operating at a frequency of 20 MHz. The pulse sequence used for encoding, diffusing and decoding the position of the water molecules (formalin) was of the type CPMG(*T*_2_)–M_z_(store)–CPMG(*T*_2_) with a period in which the magnetization of the sample (here 20 ms) is conserved along the direction of the static magnetic field and in which molecular exchange is allowed. Direct measurement of the CPMG echo train decay is performed during the last CPMG sequence (position decoding of the molecules) while the number of CPMG pulses gradually increases (position encoding). The resulting 2D spectrum is obtained by applying the two-dimensional Laplace transform [28].

**Fig. 1:**
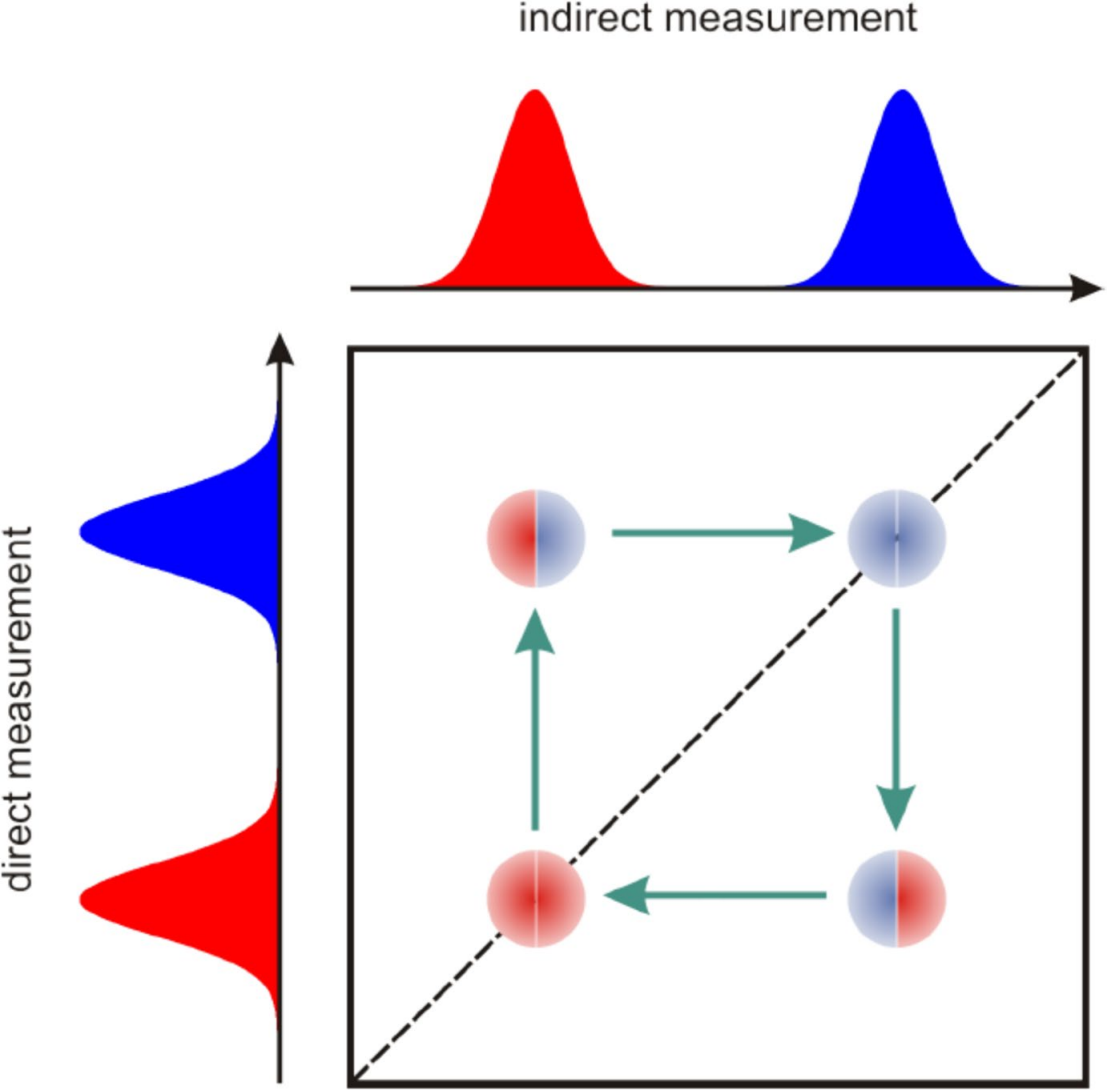
Schematic diagram of two-dimensional Laplace spectroscopy (*T*_2_-*T*_2_ exchange maps) [29]

For the interpretation of the data, the principle scheme shown in Fig. 1 is observed. For simplicity it is assumed that there are 2 hydrogen pools with different spin-spin relaxation times, *T*_2_. In the projections shown in Fig. 1, the ^1^H reservoirs (for porous biomaterials such as bone) may be represented by small pores with small *T*_2_ relaxation type which is represented with red color and the ^1^H reservoirs (larger pores in bone tissue) with larger *T*_2_ relaxation time represented with blue color. The dotted line shows the main diagonal along which peaks are expected. The colored circles inside the map are represented as two halves. The left half represents the initial encoding (in small or large pores), and the right half represents the decoding (in small or large pores) after the self-diffusion time in which fluid molecules (water/formaldehyde) can maintain or change their location.

Thus, we see that on the main diagonal we have representations of peaks with the same color: (i) red and red, if molecules were located in small pores and remained in small pores, and (ii) blue and blue, if molecules were initially located in large pores and remained in large pores. Most interesting are the extradiagonal peaks: thus, (i) a red and blue peak (left-up or above the main diagonal), for which the interpretation is that it corresponds to fluid molecules that were originally located in small pores and have diffused into large pores, and (ii) a blue-red peak, for which the interpretation is that it belongs to molecules that were originally located in large pores and have diffused into small pores. The directions of this process are also represented by clockwise arrows: it always starts from the main diagonal and goes to the main diagonal. The appearance of extradiagonal peaks is an indication that in the porous material there is an exchange process between ^1^H reservoirs with different relaxation times. Experimentally, it has been shown that there are cases where peaks on the main diagonal are missing but the^1^H reservoirs with the corresponding relaxation time is intuited by the appearance of extradiagonal peaks. This may be due to two mechanisms. The first one is related to pulse sequence parameters that are more restrictive than in the case of one-dimensional Laplace spectra (*T*_2_ relaxation time distributions) which can act as filters (especially for small *T*_2_ relaxation times). The second may be due to the fact that all fluid molecules have passed from pores of one size into pores of another size.

The experimental measurements have a finite time duration, which makes the length of CPMG pulse trains limited, leading to an exact non-localization of 2D peaks (see top right subfigure in Fig. 1), especially at large values of relaxation times, but this does not affect the integral area of peaks describing the amount of fluid with a certain dynamic behavior.

The molecular exchange shown by 2D *T*_2_-*T*_2_ EXSY (exchange spectroscopy) maps was highlighted by placing arrows on the figure highlighting the direction of the molecular exchange. Such molecular exchange maps are interpreted in terms of (i) peaks appearing along the main diagonal (see dashed line) and whose integral area is directly proportional to the number of fluid molecules that maintain their position in the same type of pore which can be, for example, a trabecular space and (ii) peaks appearing in extra-diagonal positions and indicating the presence of molecular exchange of ^1^H between reservoirs characterized by different *T*_2_ values (pores of different dimensions). The direction of this exchange (from large pores to small pores or from small pores to large pores) is indicated on the figure by arrows which are read in a clockwise (inverse trigonometric) direction. These correspond to two positions on the main diagonal. Thus, if one goes from the main diagonal down to an exchange peak-horizon and then from there to the main diagonal horizontally then the peak-horizon describes a process in which fluid molecules filling pores move from larger pores to smaller pores, and conversely, if one goes from the main diagonal up to the exchange peak-horizon and then horizontally then molecular exchange occurs from water molecules initially in narrow pores to wider pores. The presence of extradiagonal peaks indicates the existence of communication pathways between these pore types. In addition, the larger the integral area of extradiagonal peaks, the greater the amount of fluid communicating with pores at other (larger or smaller) hierarchical levels.

In a perfectly insulated system the volume of water is conserved. The MRI experiment did not provide perfect isolation, as the femoral head was enclosed in the MRI tube, but water (formaldehyde) was able to evaporate inside it, leading to elongated peaks at the top of the main diagonal indicating the presence of evaporation phenomenon. Another feature of such molecular exchange maps is that sometimes the arrows lead to positions on the main diagonal where no peaks are found (usually at extreme values of *T*_2_). This is normal and can be explained by the fact that the amount of water is small and all participate in the exchange or at even more extreme values of *T*_2_ the pulse sequence used behaves like a filter.

The advantage of the CPMG(*T*_2_)–M(store)–CPMG(*T*_2_) experiment is that it can also highlight these proton pools (water/formaldehyde) that are found in very small or very large pores. Thus, a large integral area was observed as a large area in the 2D map, or as a large amplitude in the projections of the 2D spectrum in both directions—direct and indirect—or as a large number of isosurfaces; changing color is associated with a large number of molecules. If extradiagonal peaks are involved, this indicates a more intense exchange, which is physically possible through a more permissive communication between different pore types or in other words larger spaces that can be associated with more generous trabecular spaces that are formed by decreasing bone wall dimensions.

## Results

**Fig. 2:**
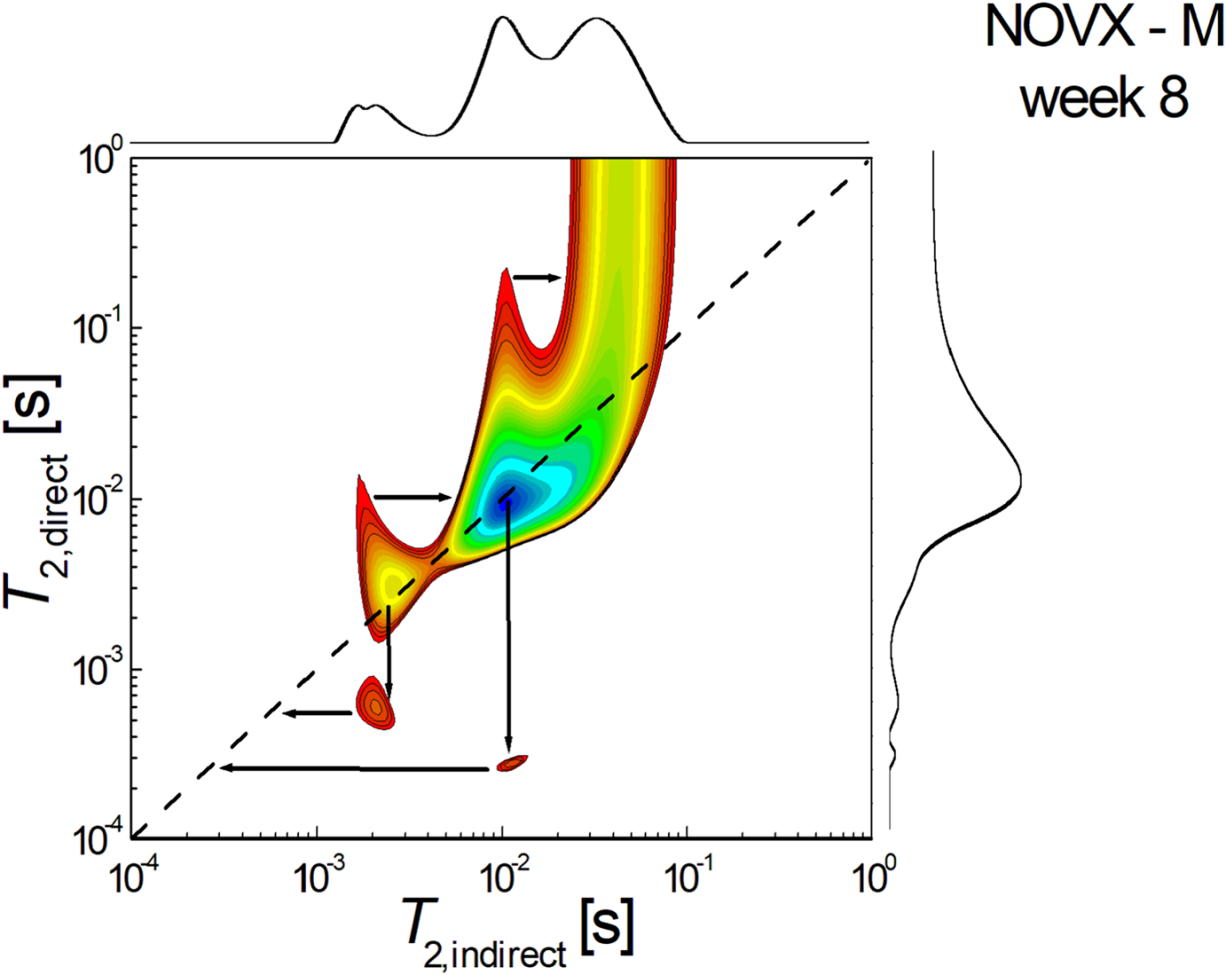
2D *T*_2_-*T*_2_ molecular exchange map measured for non-ovariectomized control rats sacrificed at week 8 after the start of treatment

Figure 2 shows a 2D *T*_2_-*T*_2_ molecular exchange map measured for non-ovariectomized control rats sacrificed at week 8 after the start of treatment. Within this map, the presence of peaks located extradiagonally indicates the molecular exchange. It can be seen that the area of the diagonally located peaks is larger than that of the extradiagonal peaks, indicating that the exchange of molecules, and therefore the connectivity of the pores, is reduced. It can be observed that there is pore connectivity with a predominant exchange from larger to smaller pores. In particular the infra-diagonal peak located at a ${T}_{2,i}\approx {10}^{-2} s \to {T}_{2,d}\approx {2.5\times 10}^{-4} s$ indicates that a relatively small number of water molecules migrated from medium pores to extra-small pores. A larger number of water molecules perform an exchange of sites between pores with more similarly dimensions (${T}_{2,i}\approx 2.5\times {10}^{-3} s \to {T}_{2,d}\approx {6\times 10}^{-4} s$). One can observe also a molecular exchange from small pores to large pores. The upper-diagonal peaks are not so well resolved (especially in the direct direction) due to the limited number of radiofrequency CPMG pulses, but the exchange can be also observed. In this sense two water exchanges are indicated: (i) ${T}_{2,i}\approx 2\times {10}^{-3} s \to {T}_{2,d}\approx {10}^{-2} s$ from small pores to medium pores and (ii) ${T}_{2,i}\approx {10}^{-2} s \to {T}_{2,d}\approx {2\times 10}^{-1} s$ from medium pore to large pore. The fact that the *T*_2_-value of ${10}^{-2} s$ (10 ms) is common means that there is a percent of water molecules which may undergo an exchange from small to large pores, but mediated by medium-sized pores. There is not directly communication between small and large pores. The extended peaks in direct dimension of *T*_2_, located at ${T}_{2,d}\approx {40\times 10}^{-2} s$, indicated that the large pores present opening to bone surface.

**Fig. 3:**
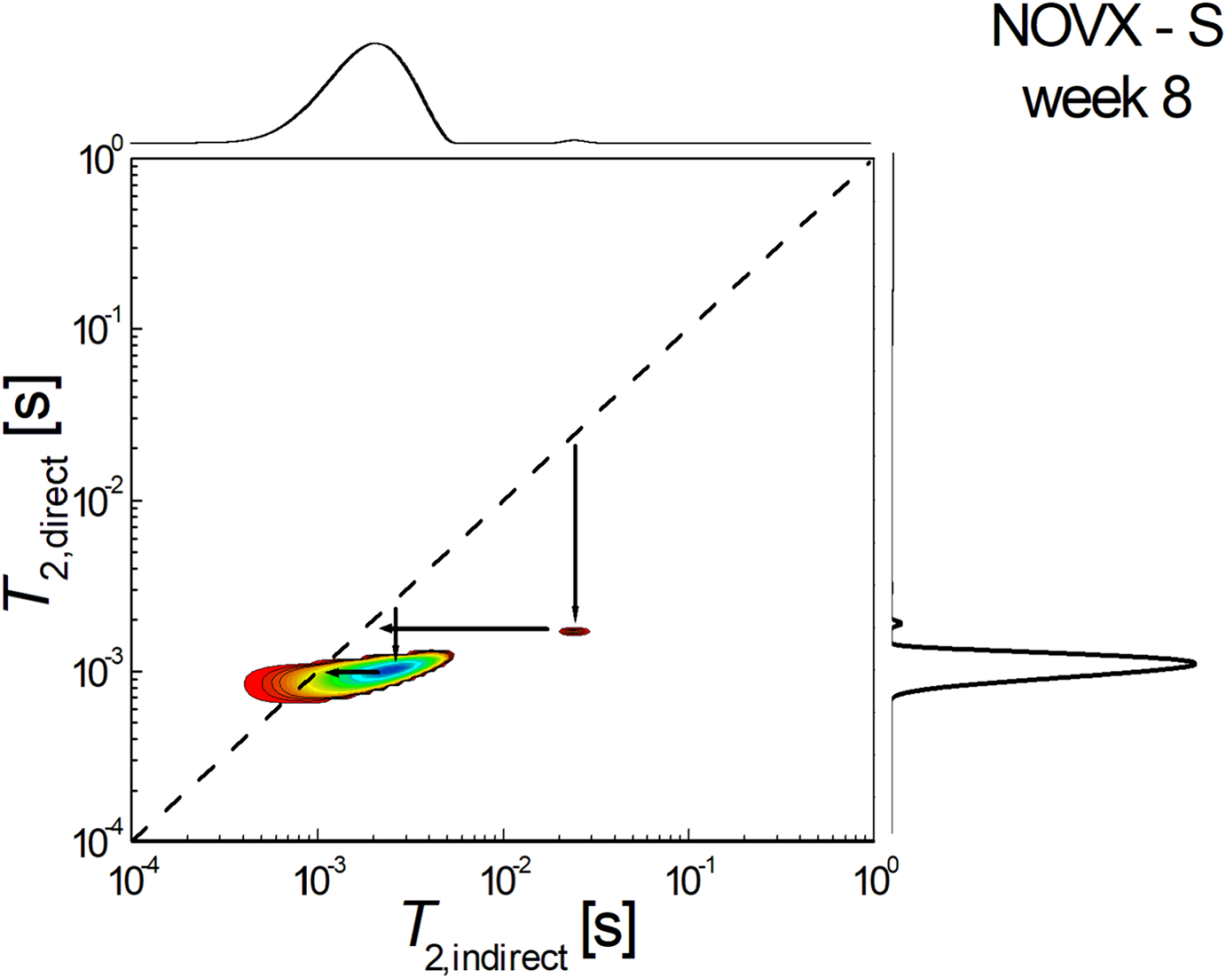
2D *T*_2_-*T*_2_ molecular exchange map measured for simvastatin-treated non-ovariectomized rats sacrificed at week 8 after the start of treatment

Figure 3 shows a 2D *T*_2_-*T*_2_ molecular exchange map measured for non-ovariectomized simvastatin treated rats sacrificed at week 8 after the start of treatment. Within this map, a significant reduction of (water in) pores can be observed through the reduced presence of diagonally located peaks. It can be seen that the area of extradiagonal peaks is reduced, which shows that the exchange of molecules, and therefore, the connectivity of the pores is also reduced. It can also be seen that there exists a connectivity of pores leading to an exchange from larger to smaller pores. In particular the infra-diagonal peak located at a ${T}_{2,i}\approx {2.5\times 10}^{-2} s \to {T}_{2,d}\approx {1\times 10}^{-3} s$ indicates that a relatively small number of water molecules migrated from medium to small pores. A larger number of water molecules perform an exchange of sites from small pores to extra-small pores, for example ${T}_{2,i}\approx 2.5\times {10}^{-3} s \to {T}_{2,d}\approx 2\times {10}^{-3} s$. As for the upper-diagonal peaks, they are missing, indicating that there is an absence of directional communication from large pores to small pores.

**Fig. 4:**
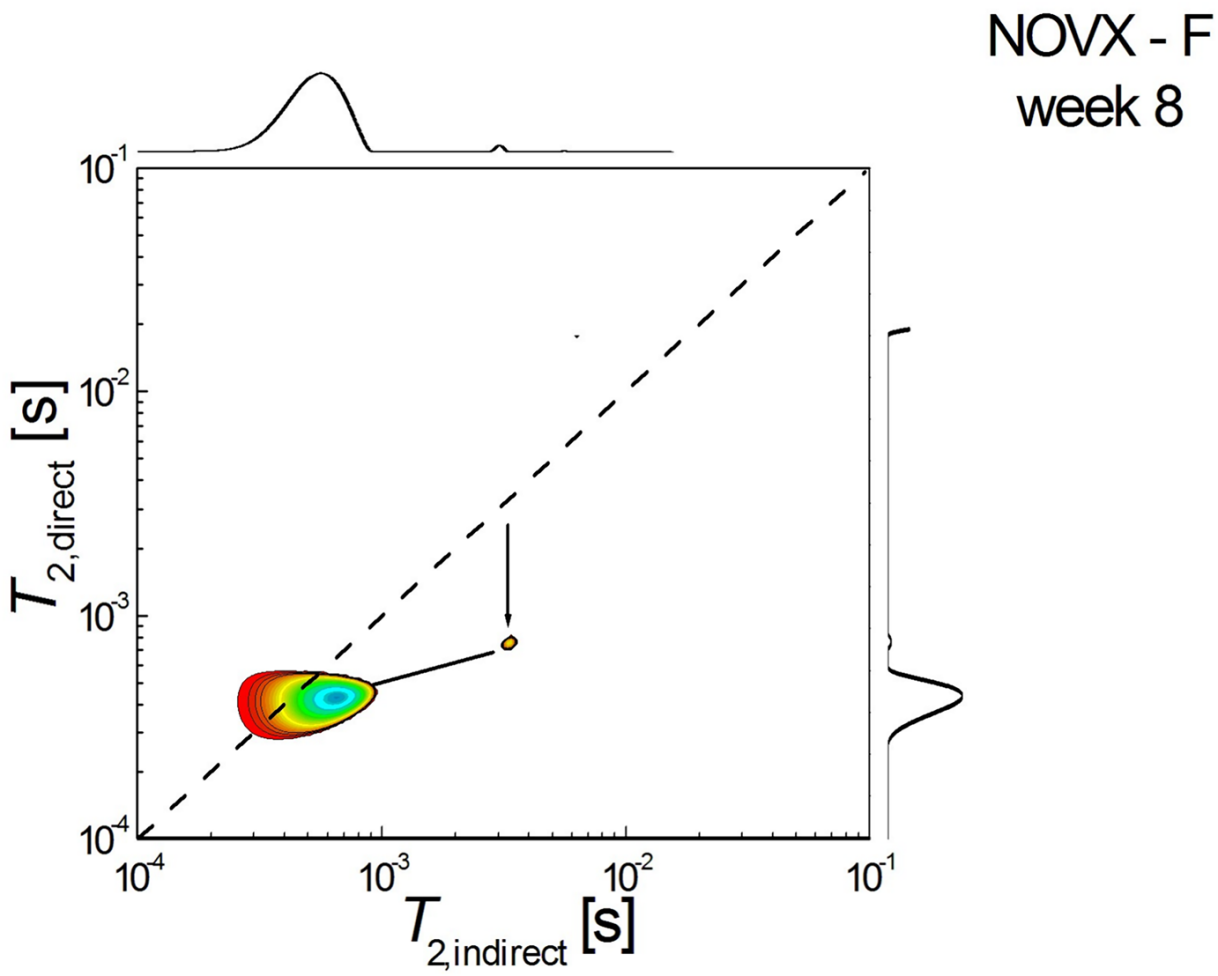
2D *T*_2_-*T*_2_ molecular exchange map measured for non-ovariectomized rats treated with fenofibrate and sacrificed at week 8 after the start of treatment

Figure 4 shows a 2D *T*_2_-*T*_2_ molecular exchange map measured for non-ovariectomized rats treated with fenofibrate and sacrificed at week 8 after the start of treatment. It can be seen that the area of extradiagonal peaks is small, which shows that the exchange of molecules, implicitly the connectivity of the pores is reduced. It can be observed that there exists pore connectivity with a predominant exchange from small to extra small pores. In particular, the infra-diagonal peak, located at ${T}_{2,i}\approx {3 \times 10}^{-3} s \to {T}_{2,d}\approx {3\times 10}^{-4}s$, indicates that a relatively small number of water molecules migrated from small pores to extra-small pores.

**Fig. 5:**
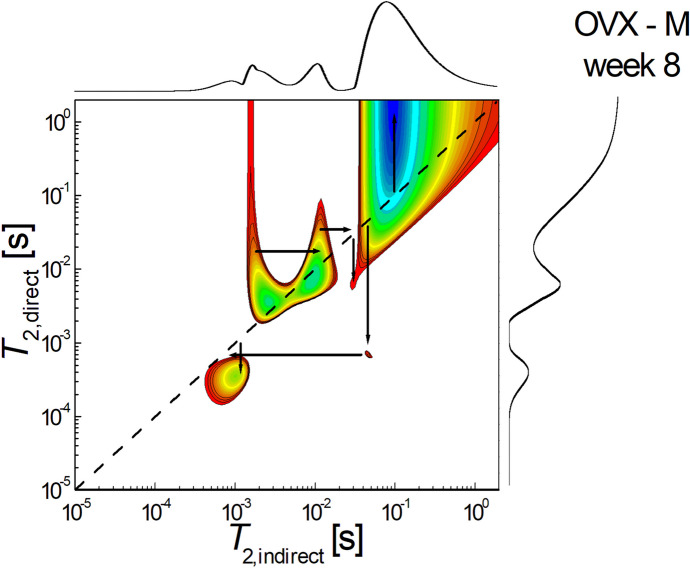
2D *T*_2_-*T*_2_ molecular exchange map measured for ovariectomized control rats sacrificed at week 8 after the start of treatment

Figure 5 shows a 2D *T*_2_-*T*_2_ molecular exchange map measured for ovariectomized control rats sacrificed at week 8 after the start of treatment. It can be seen that the area of these diagonally located peaks is smaller than that of the extradiagonal peaks, indicating that the exchange of molecules, and therefore the connectivity of the pores, is greater. It can be seen that there is pore connectivity with significant pore communication both from larger to smaller pores and in the reverse direction. In particular, the infra-diagonal peak, located at ${T}_{2,i}\approx {8 \times 10}^{-2} s \to {T}_{2,d}\approx {7\times 10}^{-4} s$, indicates that a relatively small number of water molecules migrated from medium pores to extra-small pores. Another exchange is characterized by the infra-diagonal peak, located at ${T}_{2,i}\approx {10}^{-3} s \to {T}_{2,d}\approx {2\times 10}^{-4} s$, indicating that some water molecules migrated from extra-small pores to extra-small pores. The upper diagonal peaks indicate an exchange of molecules towards larger pores with different sizes. For example, the peak, located at ${T}_{2,i}\approx {2\times 10}^{-3} s \to {T}_{2,d}\approx {2\times 10}^{-2} s,$ indicates that water molecules migrated from small pores to medium-sized pores.

**Fig. 6:**
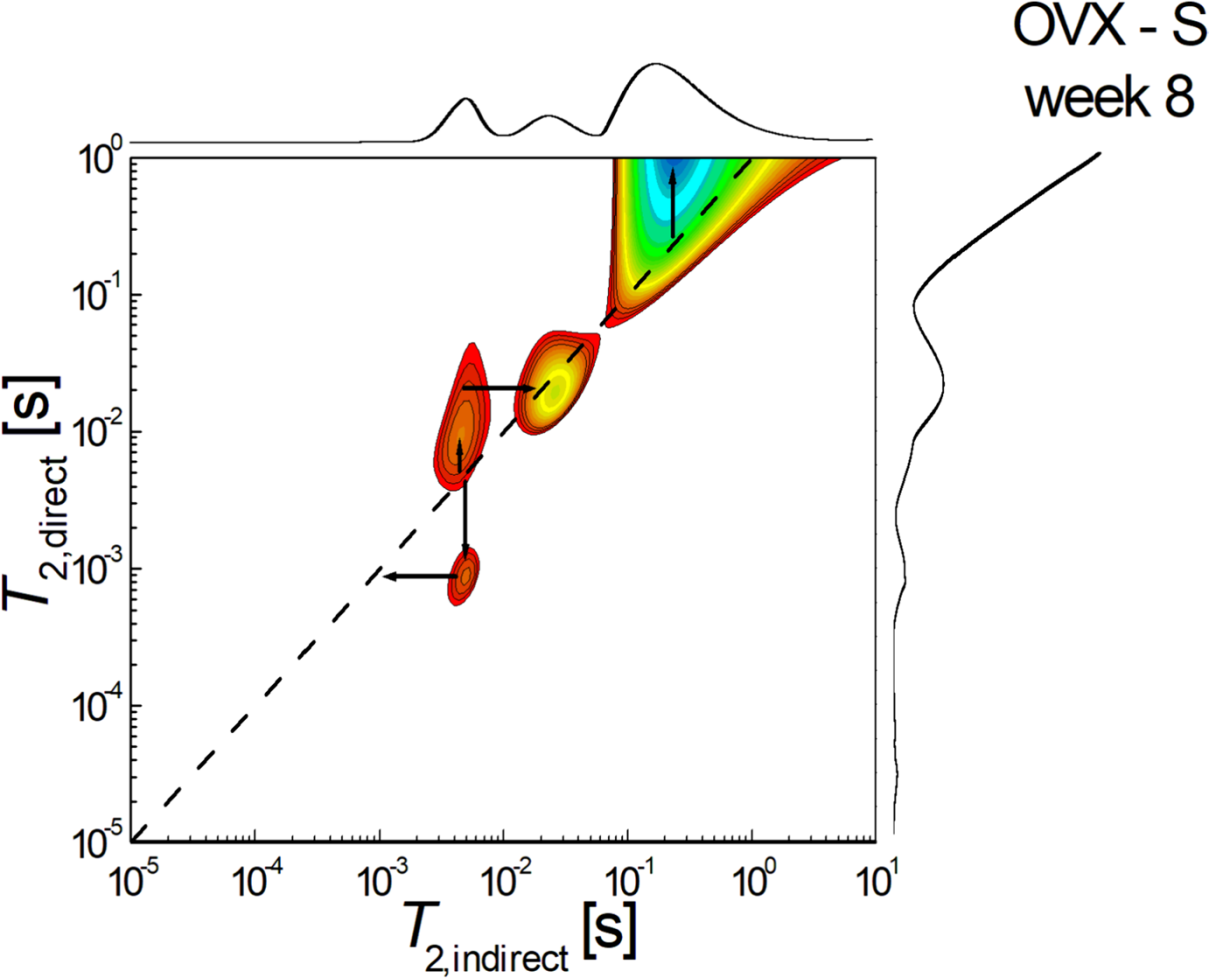
2D *T*_2_-*T*_2_ molecular exchange map measured for ovariectomized rats treated with simvastatin and sacrificed at week 8 after the start of treatment

Figure 6 shows a 2D *T*_2_-* T*_2_ molecular exchange map measured for simvastatin-treated non-ovariectomized rats sacrificed at week 8 after the start of treatment. It can be seen that the sum of the areas of extradiagonal peaks is also greater than those located on the diagonal, indicating that the exchange of molecules, and therefore the connectivity of the pores, is significant. It can be seen that there is an unbalanced pore connectivity with communication both from larger to smaller pores and in the reverse direction. The exchange can be observed via infra-diagonal peak located at ${T}_{2,i}\approx {6 \times 10}^{-3} s \to {T}_{2,d}\approx {9\times 10}^{-4} s$, which indicates that the water molecules migrated from small pores to small pores. An upper-diagonal peak for example, located at ${T}_{2,i}\approx {5 \times 10}^{-3} s \to {T}_{2,d}\approx {3\times 10}^{-2} s$, indicates that water molecules migrated from small pores to medium pores.

**Fig. 7:**
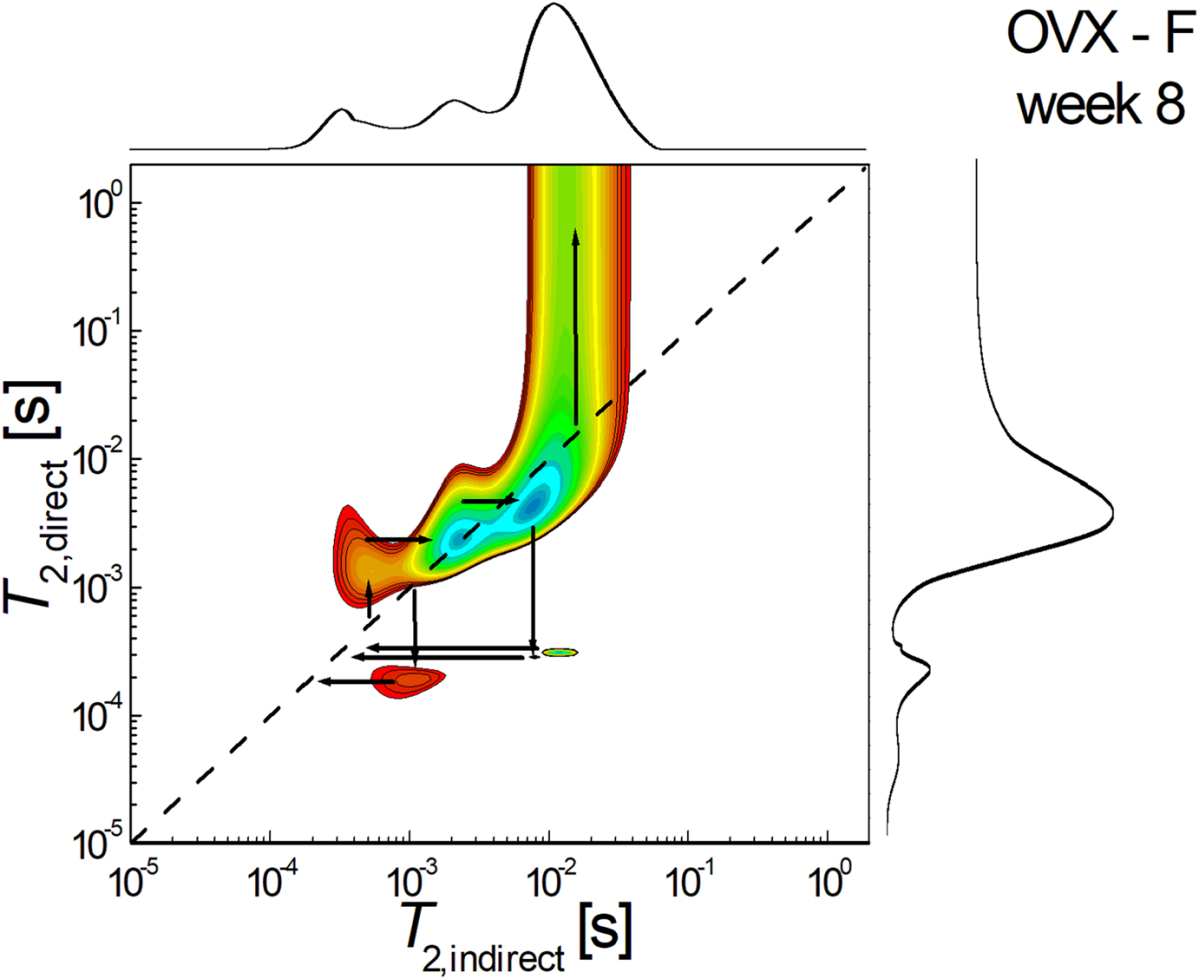
2D *T*_2_-*T*_2_ molecular exchange map measured for fenofibrate-treated ovariectomized rats sacrificed at week 8 after the start of treatment

Figure 7 shows a 2D *T*_2_-*T*_2_ molecular exchange map measured for fenofibrate-treated ovariectomized rats sacrificed at week 8 after the start of treatment. It can be seen that there is dramatically unbalanced pore connectivity with communication both from larger to smaller pores and large to small pores. However, the size of large pores is more significant and communication from large to smaller pores is predominant (${T}_{2,i}\approx {8-12 \times 10}^{-3} s \to {T}_{2,d}\approx {2-3\times 10}^{-4} s, {T}_{2,i}\approx {1\times 10}^{-3} s \to {T}_{2,d}\approx {2\times 10}^{-4} s$). The upper-diagonal peak located at ${T}_{2,i}\approx {6 \times 10}^{-4} s \to {T}_{2,d}\approx {2.5\times 10}^{-3} s$ indicates that water molecules migrated from small pores to medium-sized pores.

## Discussion

The study showed that the use of two-dimensional *T*_2_-*T*_2_ exchange maps measured by proton magnetic resonance allows the assessment of osteoporosis from the perspective of cytoarchitectonic bone tissue assessment. Thus, through the comparative evaluation of ovariectomized and non-ovariectomized control batches, it was also possible to highlight by this method the role of estrogens in the pathogenic mechanism of osteoporosis, as well as how the hypolipidemic medication represented by simvastatin and fenofibrate can influence bone tissue biology in the presence and absence of estrogens, respectively. The importance of the presence or absence of estrogens in influencing lipid-lowering medication has also been demonstrated in studies correlating results obtained by classical histological methods with those obtained by ^1^H NMR relaxometry [30]. This correlation regarding the action of hypolipidemic medication according to the presence of estrogens has also been demonstrated in relation to the fracture healing process [31, 32]. All these studies, correlated, could demonstrate the complexity of the intervention that hypolipidemic medication has on bone tissue biology.

Both the ovariectomized and non-ovariectomized control batches showed molecular exchange both from large pores to small pores and in the opposite direction from small pores to large pores. The significant difference between the two groups was that in the ovariectomized group there was a greater amount of fluid communicating through pores at other hierarchical levels, larger or smaller, supported by the larger surface area of extradiagonal peaks in this group. In the non-ovariectomized control batch, the higher amount of fluid was at the level of pores without interconnectivity. At the same time, a larger pore size could be observed in the ovariectomized control batch compared to the non-ovariectomized group. Some ^1^H NMR relaxometry studies have shown that there is a correlation in pore development in bone tissue not only in terms of the presence or absence of estrogen, but also in terms of the duration of administration of hypolipidemic medication [33].

In the non-ovariectomized group, in the two batches that received hypolipidemic treatment, there was a trend towards uniformity of pore size where interconnectivity exists by decreasing pore size. While in the non-ovariectomized control batch interconnectivity could be identified in pores that could be grouped into three categories in terms of size, in the treated batches connectivity was only evident in small pore size groups. Analysis of the two non-ovariectomized batches with hypolipidemic treatment highlighted that the fenofibrate-treated batch showed connectivity that was limited to small and very small pore sizes, while in the simvastatin-treated batch connectivity, to a lesser extent, was also maintained in medium-sized pores. Both categories of lipid-lowering treatment resulted in a significant change in bone tissue cytoarchitectonics, with the most significant change in connectivity reduction being determined by fenofibrate. These aspects of the effect of the lipid-lowering medication on the cytoarchitectonics of bone tissue in non-ovariectomized rats, correlated with the results obtained by ^1^H NMR relaxometry supporting that both simvastatin and fenofibrate have a negative effect on rat bone tissue, may complete the histological picture of the changes generated by this medication.

In contrast to the non-ovariectomized group, in the ovariectomized group, hypolipidemic treatment did not lead to a trend towards uniformity of pore size where interconnectivity exists. In this group it was possible to identify, according to pore size, four categories of pores in the control and fenofibrate-treated batch, while in the simvastatin-treated batch it was not possible to show interconnectivity between the smallest pore size group. This trend of preservation of bone tissue cytoarchitectonics correlated with the evidence obtained by ^1^H NMR relaxometry [30] may support, based on two distinct methods, the idea that both simvastatin and fenofibrate can reduce osteoporosis caused by estrogen deprivation caused by ovariectomy.

Analyzing pore size and pore interconnectivity in the ovariectomy group, if one compares the batch that received simvastatin treatment with the control batch, it can be seen that the simvastatin treated batch showed not only a decrease in pore size but also a reduction in interconnectivity between pores. On the other hand, in the ovariectomized batch treated with fenofibrate, it could be observed that the cytoarchitectonics of the bone tissue is similar in the structure of the molecular exchanges with the control batch, especially with the non-ovariectomized control batch. This distinct effect on cytoarchitectonic change, if correlated with evidence from studies using visible spectroscopy, demonstrated that in ovariectomized rats fenofibrate treatment promotes the callus formation process, whereas simvastatin delays this process [34], which could explain why primary callus remodeling is slower in ovariectomized and simvastatin-treated rats.

The osteoprotective potential of a drug would consist not only in increasing bone density by reducing intertrabecular pores, but also in preserving or optimizing the cytoarchitectonics of bone tissue. Based on comparative data between the results recorded using two-dimensional *T*_2_-*T*_2_ maps measured by proton NMR for the evaluation of the ovariectomized control batch and the ovariectomized fenofibrate-treated batch, respectively, a potential osteoprotective effect of this drug could be observed [27].

Comparative analysis of the results recorded using two-dimensional *T*_2_-*T*_2_ maps in the non-ovariectomized control batch and the ovariectomized batch treated with fenofibrate showed similar bone tissue cytoarchitectonics. It can be seen that the area of diagonally located peaks is larger than that of the extradiagonal peaks, indicating that the exchange of molecules and therefore the connectivity of the pores is reduced and that the treatment with fenofibrate further reduces this interconnectivity. In terms of interconnectivity, in the non-ovariectomized control batch, it can be observed that there is a pore connectivity with a predominant communication from larger pore sizes to smaller pore sizes; a similar aspect can be observed in the ovariectomized batch treated with fenofibrate. By correlating the information obtained from the registration of two-dimensional *T*_2_-*T*_2_ exchange maps with the evidence obtained from ^1^H NMR relaxometry [30], it can be stated that the interconnection of this information allows a better assessment of bone porosity from the perspective of the risk of developing pathological fractures.

## Conclusions

Hypolipidemic medication influences bone tissue cytoarchitectonics in both the presence and absence of estrogen. While in the presence of estrogen, the hypolipidemic drug alters the molecular changes observed in healthy bone tissue in the EXSY T2-T2 maps, in the absence of estrogen, the hypolipidemic drug attenuates the osteoporotic effects caused by the absence of this hormone. The molecular exchange map showed that treatment with fenofibrate administered in osteoporosis results in a bone structure with pores showing morphology and connectivity similar to non-osteoporotic/healthy bone tissue.

## Data Availability

No datasets were generated or analyzed during the current study.

## Declarations

### Disclaimer

The authors confirm that the work described has not been published, that it is not under consideration for publication elsewhere, that its publication has been approved by all co-authors, and that its publication has been approved (tacitly or explicitly) by the responsible authorities at the institution where the work is carried out.
